# Synergistic antitumor effect of puerarin combined with 5-fluorouracil on gastric carcinoma

**DOI:** 10.3892/mmr.2014.3016

**Published:** 2014-11-27

**Authors:** XU-FENG GUO, ZI-RONG YANG, JUN WANG, XIAO-FEI LEI, XIAO-GUANG LV, WEI-GUO DONG

**Affiliations:** 1 Department of Gastroenterology, Renmin Hospital of Wuhan University, Wuhan, Hubei 430060, P.R. China; 2 Key Laboratory of Hubei Province for Digestive System Disease, Wuhan, Hubei 430060, P.R. China

**Keywords:** chemotherapy, 5-fluorouracil, puerarin, gastric carcinoma, side effect

## Abstract

Combination chemotherapy is a crucial method in the treatment of gastric cancer. The aim of the present study was to investigate the inhibitory effects of puerarin and 5-fluorouracil (5-FU) on BGC-823 gastric cancer cells *in vitro* and *in vivo*. The *in vitro* growth inhibition of puerarin or 5-FU alone or combined on BGC-823 cells was determined using a cell counting kit 8 (CCK-8) on living cells. Apoptotic morphological features and proteins expression levels were detected by Hoechst 33258 staining, an Annexin V/propidium iodide apoptosis kit and western blot analysis, respectively. Tumor xenografts were established in nude mice and the inhibitory effects and side effects were detected. Results of the CCK-8, Hoechst 33258 staining and flow cytometry revealed that the combined treatment was more effective than the separate treatments. The tumor volume was 90.65% of that of the controls and the mean tumor weight was only 0.125 g at the end of the experiment in the combination group compared with the control group (0.822 g). In addition, it was determined that liver and renal toxicity did not increase in combined treatment. These findings showed that puerarin and 5-FU produced a significant synergic effect on gastric cancer cells, while there was no increase in side effects.

## Introduction

Gastric cancer (GC) is the fourth most frequent type of cancer around the world (1). In East Asia, it is the second leading cause of cancer-associated mortalities, and ~934,000 new patients are diagnosed with gastric cancer each year (2). In spite of advanced chemotherapy and surgical resection, the majority of patients are already at the advanced stage at diagnosis, negating the use of a surgical operation. Patients with an identical tumor, nodes and metastasis stage may even have different prognoses (3). Therefore, the application of novel chemotherapeutic agents and combinations of regimens may provide an improved therapy for advanced GC and reduce the toxicity of these drugs.

The compound 5-fluorouracil (5-FU) remains a first line antineoplastic treatment in clinical practice (4). However, GC cells are becoming resistant to existing chemotherapeutic compounds, including 5-FU. Therefore, it is necessary to explore novel chemotherapeutic regimens for the treatment of advanced GC including metastatic GC. Puerarin [8-(β-d-glucopyranosyl-7-hydroxy-3-(4-hydroxyphenyl)-4-H-1-benzopyran-4-one], with the molecular formula C_21_H_20_O_9_, was isolated from the herbal medicine Radix puerariae (5,6). Previous studies have shown that puerarin has beneficial effects on cardiovascular, neurological and hyperglycemic disorders (7–9). Researchers found that puerarin has an anticancer effect on HT29 cells and enhances the antiproliferative effects of other antineoplastic agents (10,11). However, to the best of our knowledge there have been no studies on the effectiveness of puerarin on GC cells, and the mechanism of its protective effects has remained elusive.

The aim of the present study was to evaluate the combined effects of puerarin and 5-FU on BGC-823 gastric carcinoma cells *in vitro* and *in vivo*.

## Materials and methods

### Cells and reagents

The BGC-823 human gastric cancer cell line was purchased from the Shanghai Cell Collection (Shanghai, China). The cells were maintained in DMEM (Gibco-BRL Gaithersburg, MD, USA) supplemented with 10% heat-inactivated fetal bovine serum (FBS) and 1% antibiotic solution (penicillin 100 U/ml and streptomycin 100 g/ml; Beyotime, Shanghai, China) at 37°C in a humidified atmosphere of 95% air/5% CO_2_. Puerarin and 5-FU were obtained from Sigma-Aldrich (P5555, F6627; St. Louis, MO, USA) and were 98% pure by reverse-phase high-performance liquid chromatography.

### Estimation of cell proliferation

Cell proliferation was quantified with a Cell Counting kit-8 assay (CCK-8; Dojindo, Shanghai, China). Cells were suspended in a complete DMEM and subsequently seeded in 96-well microtiter plates at a density of 1×10^5^ per well. Following exposure to puerarin (400, 800, 1600, 3200 or 6400 μM), 5-FU (20, 40, 80, 160 or 320 μM) or puerarin+5-FU for 48 h, cells were incubated at 37°C for another 4 h with CCK-8 (10 μl per well). Absorbance was measured at a wavelength of 490 nm using an iMark microplate reader (Bio-Rad, Hercules, CA, USA). Each sample was subjected to three independent experiments.

### Evaluation of the combined effects of puerarin and 5-FU

As previously described (12,13), the following equation was used to evaluate the nature of the interaction between puerarin and 5-FU: D = D_m_ [fa/(1-fa)]^1/m^, where D is the dose, D_m_ is the median effect dose required to produce (analogous to the IC_50_), fa is the fraction of the system affected by D, and m is a Hill-type coefficient signifying the sigmoidicity of the dose-effect curve. The combination index (CI) was obtained by using the Biosoft CalcuSyn software (Biosoft Version 2.0, Cambridge, UK) written in BASIC for automatic graphing of the CI with respect to fa. When smaller than 1, equal to 1, or greater than 1, CI indicates synergism, summation, antagonism, respectively.

### Hoechst asssay

Detection of apoptotic morphological features was performed using Hoechst 33258 staining (Beyotime). Cells were cultured in a six-well plate and incubated with puerarin (1,600 μM), 5-FU (80 μM) or puerarin+5-FU for 48 h. Then, 20 μM Hoechst reagent was added to each well with for 10 min at room temperature. Changes in nuclear morphology were observed with a fluorescence microscope (BX53F, Olympus, Tokyo Japan). In each group, ten microscopic fields were selected randomly and counted.

### Annexin V/propidium iodide (PI) staining

An Annexin V/PI apoptosis kit was used to quantify the percentage of cells undergoing apoptosis (Multisciences, Hangzhou, China). Cells were incubated for 48 h with either puerarin, 5-FU or puerarin and 5-Fu. Following incubation, the cells were washed twice with cold phosphate-buffered saline and resuspended in binding buffer (500 μl) at a concentration of 1×10^6^ cells/μl. Then, 5 μl Annexin V-fluorescein isothiocyanate and 10 μl PI were added and the cells were incubated at room temperature for 5 min in the dark. At the end of the incubation period, cell apoptosis was analyzed on a FACScan flow cytometer (Becton Dickinson, Franklin Lakes, NJ, USA).

### Western blot analysis

A wet transfer system was used to detect the protein levels. Proteins were resolved by SDS-PAGE (10%, Beyotime) and transferred onto nitrocellulose membranes (Bio-Rad). Membranes were blocked with 10% non-fat dry milk and incubated with primary antibodies (Bcl-2, Bax, GAPDH mouse monoclonal antibodies; 1:1,000; Santa Cruz Biotechnology, Inc., Dallas, TX, USA) overnight at 4°C. The following day, horseradish peroxidase-conjugated polyclonal goat anti-rat secondary antibodies (1:2,000; Santa Cruz Biotechnology, Inc.) were added, and the cells were incubated for 2 h at room temperature. Then, the membranes were placed in an enhanced chemiluminescence system (Millipore, Bedford, MA, USA) and imaged in a dark room. The antibodies were used as follows: B-cell lymphoma-2 (Bcl-2), Bcl-2-associated X (Bax) and GAPDH (1:1,000, Santa Cruz Biotechnology, Austin, TX, USA).

### Tumor xenograft experiment in nude mice

Male BALB/c-nu/nu nude mice, [weighing 16–18 g, 4–6 weeks of age, purchased from the Center of Experimental Animals of Wuhan University, (Wuhan, China)] were caged in groups of six and fed with a standard diet and water ad libitum. The study was approved by the ethics committee of Remnin Hospital of Wuhan University. Cells (5×10^6^ cells/mouse) were injected subcutaneously into the right dorsal area. When tumors grew to a mean volume of 160 mm^3^, the mice were treated were as follows: *i*) Control, 0.9% saline; *ii*) puerarin group, 30 mg/kg/day puerarin, *iii*) 5-FU group, 12 mg/kg/day 5-FU; *iv*) puerarin+5-FU group, combination of puerarin (30 mg/kg/day) and 5-FU (12 mg/kg/day), three times a week for three weeks. Tumor volume (TV) and weight were recorded every three days. TV was calculated by the following formula: TV (mm^3^) = d^2^ × D/2, where d and D are the shortest and longest diameters, respectively. Following three weeks of treatment, the mice were sacrificed by orbital sinus bleeding and the tumors were segregated and weighed. Liver and renal injury were measured through detection of the levels of alanine aminotransferase (ALT), aspartate aminotransferase (AST), blood urea nitrogen (BUN) and serum creatinine (Cr). The tumor tissues were harvested for pathological examination to confirm that they were the same type of tumor. The levels of apoptosis in the cells of the tumor xenograft were measured by terminal deoxynucleotidyl transferase dUTP nick end labeling (TUNEL) assay according to the manufacturer’s instructions (Roche Diagnostics, Branchburg, NJ, USA).

### Statistical analysis

Statistical analyses were performed using SPSS software version 17.0 (SPSS, Inc., Chicago, IL, USA). All data are expressed as the mean ± the standard deviation. The mean values of the different groups were compared using non-parametric analysis (Mann-Whitney rank sum test), and a value of P<0.05 was considered to be statistically significant.

## Results

### Cytotoxic effects of puerarin and 5-FU on BGC-823 cells

**Figure 1:**
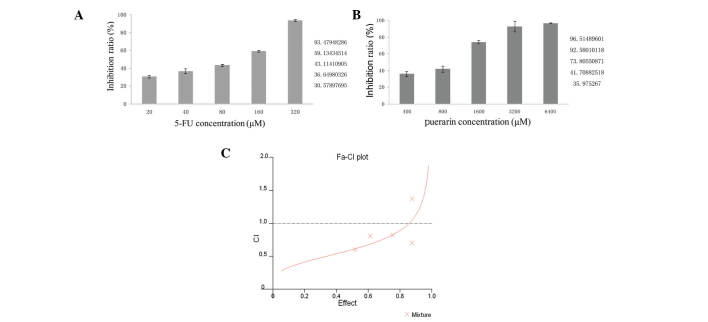
Effect of puerarin or 5-FU alone on BGC-823 cells. The proliferation of BGC-823 cells was inhibited by (A) 5-FU and (B) puerarin in a dose-dependent manner (P<0.01 compared with 20 μM 5-FU and 400 μM puerarin, respectively). (C) The CI values were determined by the method by Chou and Talalay. As shown here, the CI values are <1 when the fractions affected are <0.8775. Thus, puerarin and 5-FU at certain concentrations had synergistic effects in inhibiting the proliferation of BGC-823 cells. 5-FU, 5-fluorouracil; CI, combination index; fa, fraction of the system affected by the dose.

After 48 h of drug exposure, growth of BGC-823 cells was clearly inhibited in a dose-dependent manner *in vitro*. As shown in Fig. 1, 20 μM 5-FU had an inhibition rate of 30.58%, which increased to 93.48% at 320 μM (Fig. 1A). The inhibition rates caused by puerarin were 35.90% (400 μM) and 96.51% (6,400 μM; Fig. 1B). Combined treatment with these two drugs significantly decreased the viability of BGC-823 cells compared with the effect of either drug used alone.

### Synergistic effect of puerarin and 5-FU on BGC-823 cell growth

Following exposure to the drugs for 48 h, the inhibition rate was tested. The combination effects were studied by the method of Chou and Talalay (12). Treatment with 2,400 μM puerarin or 240 μM 5-FU produced nearly 75% growth inhibition when used alone. The same inhibition rate was achieved with a combination of 1,600 μM puerarin and 80 μM 5-FU (20:1). Therefore, puerarin was able to decrease the efficient dose of 5-FU by two-fold. The experiments were repeated independently in triplicate. The CI value was <1 when the fractions affected were <0.8775, as shown in Fig. 1C, which indicated that puerarin and 5-FU had a synergistic effect on inhibiting BGC-823 cells.

### Apoptosis induced by puerarin and 5-FU

**Figure 2:**
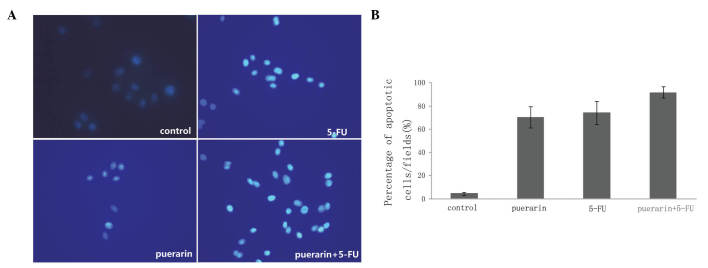
Puerarin and 5-FU either in combination or alone induce apoptosis in BGC-823 cells. (A) Detection of apoptotic morphological changes by Hoechst 33258 staining. Following exposure to puerarin (1,600 μM), 5-FU (80 μM) or puerarin+5-FU for 48 h, BGC-823 cells were incubated with 20 μM Hoechst 33258 for 10 min at room temperature. Then apoptotic features were assessed by observing chromatin condensation and fragment staining. (B) Quantification of results. ^*^P<0.05 compared with the control group and ^#^P<0.05 compared with peurarin or 5-FU. Magnification, ×400. 5-FU, 5-fluorouracil.

**Figure 3:**
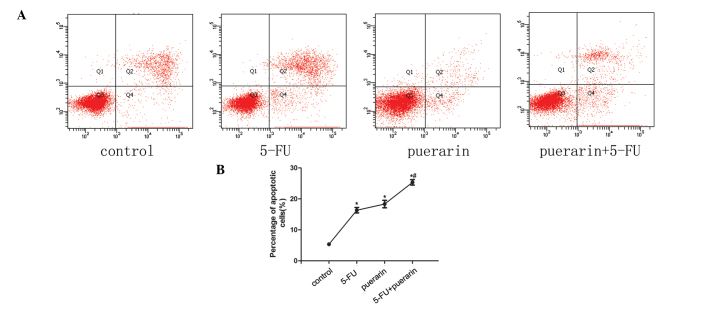
Annexin V/PI apoptosis kit was used to quantify the percentage of cells undergoing apoptosis (X axis, Annexin V; Y axis, PI). (A) Flow cytometry results. (B) Percentage of apoptotic cells in each group. Puerarin combined with 5-FU or used individually significantly promoted apoptosis compared to the control group (^*^P<0.05), and the combined effect was stronger than the effect of puerarin or 5-FU alone (^#^P<0.05). 5-FU, 5-fluorouracil; PI, propidium iodide.

Apoptosis was one of the predominant types of programmed cell death which involved a series of biochemical events leading to specific cell morphology characteristics, including cell shrinkage, nuclear fragmentation, chromatin condensation and chromosomal DNA fragmentation. As shown in Fig. 2A and B, nuclear fragmentation and chromatin condensation in the puerarin + 5-FU group were brighter than in the groups treated with one of the drugs alone (P<0.05) and the control group (P<0.05). BGC-823 cells were treated with low concentrations of puerarin (1,600 μM) and 5-FU (80 μM) for 48 h alone or in combination, respectively. The Annexin V/PI apoptosis kit was used to examine the proportion of apoptotic cells. Data indicated that the combined effect was greater than the effects of puerarin and 5-FU individually (P<0.01, Fig. 3).

### Mitochondrial pathway protein expression by western blot

**Figure 4:**
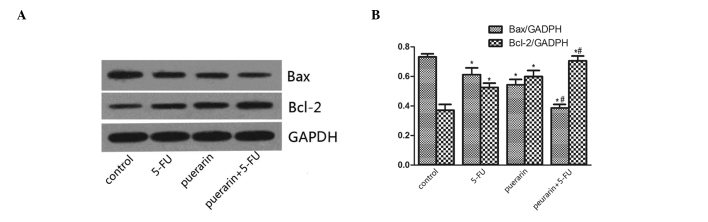
Apoptosis induced by puerarin and 5-FU alone or in combination in BGC-823 cells. (A) Western blotting analysis of Bax and Bcl-2 protein following exposure to puerarin (16,000 μM) and 5-FU (80 μM) alone or in combination; (B) Analysis of the expression of proteins. ^*^P<0.05 compared with the control group and ^#^P<0.05 compared with 5-FU or puerarin. 5-FU, 5-fluorouracil; Bcl-2, B-cell lymphoma 2; Bax, Bcl-2-associated X.

In order to verify the apoptotic mechanism, the levels of the mitochondrial pathway proteins Bax and Bcl-2 were detected. As shown in Fig. 4, the Bax/GAPDH protein expression levels were increased in the puerarin and 5-FU treatment groups. By contrast, the Bcl-2/GAPDH protein expression levels were reduced. Results showed that the effect of combined treatment was greater than that of individual treatment.

### Effect of puerarin and 5-FU on tumor development in vivo

**Figure 5:**
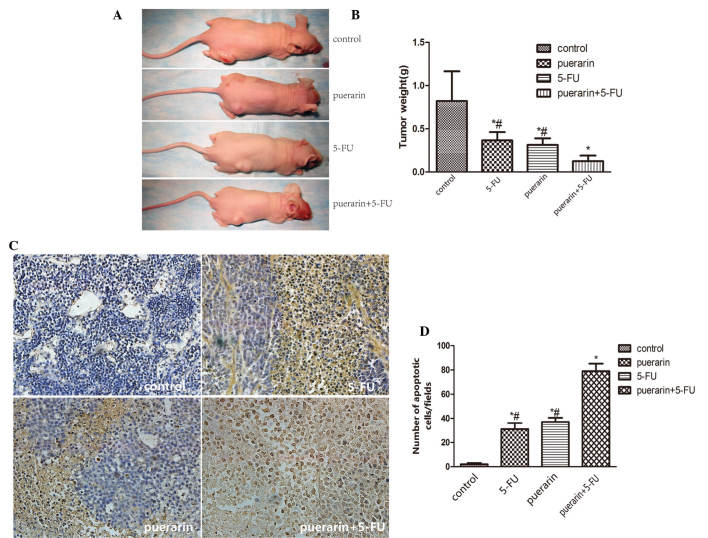
(A) Dimensions and (B) weight of tumors at the end of the experiment. (C) Apoptotic cells were detected in xenograft tumor tissue using the TUNEL assay. Apoptotic cells appear brown and are indicated by arrows. Magnification, ×200. (D) Quantified results of the TUNEL assay. Values are presented as the mean ± standard deviation (n=6 per group). ^*^P<0.05, compared with the puerarin+5-FU group; ^#^P<0.05, compared with the control group. 5-FU, 5-fluorouracil; TUNEL, terminal deoxynucleotidyl transferase dUTP nick end labeling.

The effect of puerarin and 5-FU on the growth of primary tumor xenografts in nude mice was examined. Tumor volume was recorded every three days. The volumes of the tumors in the treatment groups were clearly reduced compared with those in the control group, while the inhibition rate in the combination group was 90.65%, which was a more significant inhibition than that in the other three groups (P<0.05; Table I). The data showed that the effect of the combined treatment was superior to the effect of puerarin or 5-FU individually. The mean tumor weight in the combination group was only 0.125 g at the end of the experiment compared with the control group (0.822 g) (Fig. 5A and B). TUNEL assays of the subcutaneous tumor tissue sections demonstrated that puerarin combined with 5-FU produced clear cell apoptosis in the tumor mass, while little apoptosis was observed in the control group (P<0.05, Fig. 5C and D).

### Evaluation of side effects

At the end of the experiment, 24 mice were necropsied to assess the side effects. There were no injuries to the liver or kidney observable with the naked eye. ALT, AST, BUN and Cr levels in the serum were detected to evaluate liver or renal injuries. The results were not indicative of any injury and there were no significant variations among the four tested groups (P>0.05, Table II).

## Discussion

GC remains the most commonly diagnosed type of cancer worldwide, exhibiting a high annual mortality (2,3). Early diagnosis and combined chemotherapies have increased the survival rate. Numerous chemotherapeutic agents, including 5-FU, cisplatin, doxorubicin and paclitaxel, have been used for clinical treatment of patients for years. However, the overall outcome remains poor due to drug resistance, radiotherapy or side effects. Studies have shown that the combination of Chinese herbs with these drugs enhanced the efficacy of therapy of human tumors, including gastric cancer (5). Therefore, combination chemotherapy has become one of the most important means of improving survival of gastric cancer patients.

5-FU, which is widely used for the treatment of several human malignancies, has been used in clinical treatments for years (14). However, administration of 5-FU alone often leads to undesirable side effects and drug resistance. In addition, 5-FU has been used in combination with other agents, which resulted in fewer side effects, superior pharmacological properties and an improved antitumor effect (15–18). Therefore, it is necessary to identify novel agents enhancing the anticancer effect of 5-FU. Recently, traditional medicines have been exploited to treat tumors, particularly in China. Puerarin (isolated from a Chinese Medicinal herb), has been used in China for years. However, little is known about the effects of puerarin on GC cells. In the present study, the inhibitory effect of puerarin combined with 5-FU on GC was investigated *in vitro* and *in vivo*.

The results of the present study revealed that puerarin or 5-FU alone significantly inhibited the proliferation of BGC-823 cells in a dose-dependent manner (puerarin, 400–6,400 μM; 5-FU, 20–320 μM). These results showed that puerarin combined with 5-FU at appropriate concentrations had a synergistic effect on BGC-823 cells, compared with that of puerarin or 5-FU alone. Wang *et al* (11) examined the effect of puerarin on HT-29 cells, and their results showed that puerarin, with a higher tolerance *in vitro*, inhibits the growth of HT-29 cells by the induction of early apoptosis. A study by Wang *et al* (19) showed that using puerarin reversed multidrug resistance (MDR) in a nude mouse model of human GC and reduced the expression of P-glycoprotein and multidrug resistance protein (19). Hien *et al* (20) found that puerarin downregulated MDR1 expression via nuclear factor κ-B and CRE transcriptional activity-dependent upregulation of 5′ adenosine monophosphate-activated protein kinase in MCF-7/adr cells. Furthermore, a study by Han *et al* (21) indicated that puerarin increased Bax expression and reduced Bcl-2 expression, inhibiting the cell cycle in G0/G1 phase. In the present study, the results of the flow cytometric analysis showed that puerarin and 5-FU inhibited the early apoptosis of BGC-823 cells, with a synergistic anticancer effect observed when puerarin and 5-FU were used together. The mechanism of this synergistic effect of puerarin (1,600 μM) and 5-FU (80 μM) on BGC-823 cells was investigated by western blot analysis. The results demonstrated that the cytotoxic effects of 5-FU were potentiated by the addition of puerarin. The side effects of puerarin and 5-FU were also observed *in vivo*. Serological indicators were not significantly different between combined or single treatment and control groups.

In conclusion, the present study indicated that low-dose 5-FU combined with puerarin inhibited cell viability more effectively, compared with 5-FU and puerarin alone. Puerarin may potentiate the antiproliferative effect of 5-FU and reduce the therapeutically required dose of 5-FU, without increasing the toxicity, which provided a valuable novel treatment for patients with GC.

## Figures and Tables

**Table I:** Inhibitory effects of puerarin and 5-FU on BGC-823 tumor volume in nude mice.

Group	n	Volume (mm^3^)	Inhibition rate (%)
Puerarin	6	236.02±40.50^a^,^b^	68.37
5-FU	6	194.70±35.33^a^,^b^	73.90
Puerarin+5-FU	6	69.76±24.01^b^	90.65
Control	6	746.10±114.56	

Data are presented as the mean ± standard deviation (six mice per group) and expressed as inhibition rate (%)=[1-mean of tumor volume from the experimental group/mean of tumor volume for the control]x100%.

a P<0.05, significantly different from the puerarin+5-FU group;

b P<0.05, significantly different from the control group.

5-FU, 5-fluorouracil.

**Table II:** Effect of puerarin combined with 5-FU or alone on hepatic and renal function.

Group	n	ALT (U/l)	AST (U/l)	Urea (μmol/l)	Cr (μmol/l)
Puerarin	6	34.33±4.72	131.50±12.88	7.25±2.50	17.69±2.48
5-FU	6	33.67±3.44	129.17±26.63	6.96±1.29	15.72±1.29
Puerarin+5-FU	6	35.50±10.37	136.33±17.50	7.34±0.53	19.21±5.59
Tumor control	6	31.67±10.56	124.50±22.49	7.01±0.44	15.81±2.91
Normal control	6	32.17±8.70	130.17±15.08	7.04±0.81	16.62±2.48

Values are presented as the mean ± standard deviation (SD), with n=6 mice/group. Groups were treated as follows: Puerarin (30 mg/kg/day), 5-FU (12 mg/kg/day), puerarin (30 mg/kg/day)+5-FU (12 mg/kg/day), tumor control (saline of equal volume) or normal control (no injection). No differences were observed in serum ALT, AST, urea or Cr among the groups (P>0.05). ALT, alanine aminotransferase; AST, aspartate aminotransferase; Cr, serum creatinine; 5-FU, 5-fluorouracil.
